# Radially Based Extensor Retinacular Sling Reconstruction for Extensor Carpi Ulnaris Subsheath Injuries

**DOI:** 10.1016/j.jhsg.2022.11.003

**Published:** 2022-12-07

**Authors:** Michael A. Mastroianni, Matthew Leibman, Mark Belsky, Mark A. Vitale, David E. Ruchelsman

**Affiliations:** ∗ NewYork Presbyterian/Columbia University Irving Medical Center, New York, NY; † Newton-Wellsley Hospital, Hand Surgery PC, Newton, MA; ‡ Greenwich Hospital, Orthopaedic & Neurosurgery Specialists, Greenwich, CT; § Newton-Wellesley Hospital/Mass General Brigham, Newton, MA

**Keywords:** ECU subsheath, Extensor retinacular sling, TFCC, Ulnar-Sided wrist pain, Ulnocarpal compartment

## Abstract

**Purpose:**

Extensor carpi ulnaris (ECU) subsheath injuries result in ulnar-sided wrist pain and often present concurrently with intrinsic ECU pathology and ulnocarpal compartment injuries. There is a lack of surgical outcome data despite the variety of described ECU subsheath pathologies and reconstructive strategies.

**Methods:**

We retrospectively reviewed our hand-center experience of 33 patients who prospectively underwent radially based extensor retinacular sling ECU subsheath reconstruction by 4 hand surgery-fellowship-trained surgeons between April 2010 and April 2021. Preoperative clinical and magnetic resonance imaging findings, along with intraoperative findings, were cataloged. Statistical analysis was conducted via a 2-tailed paired *t* test.

**Results:**

The median age at the time of surgery was 44 years (range, 18–63 years). Twenty (60.6%) patients underwent reconstruction on their dominant wrist. The median time between symptom onset and surgery was 6.5 months (range, 4 days–16.1 years). Eight (18%) patients were collegiate-level or professional athletes. Ten (30.3%) patients had frank ECU snapping on the preoperative examination with no recurrence or apprehension on the postoperative examination. All 33 patients underwent a preoperative magnetic resonance imaging. Fifteen (45.4%) patients had intrinsic ECU tendinopathy, 19 (57.6%) patients had ECU tenosynovitis, 18 (54.5%) patients had triangular fibrocartilage complex tears, 20 (60.6%) patients had ulnocarpal synovitis, and 2 (6.1%) patients had lunotriquetral interosseous ligament tears. The mean postoperative pain on a visual analog scale was 0.39 ± 0.55. Grip strength, wrist flexion-extension, and pronosupination arcs (*P* < .05) showed excellent recovery after surgery. The mean time to unrestricted return to sports was 97.3 ± 19.7 days for the athletes in this study. There were no major complications.

**Conclusions:**

Radially based extensor retinacular sling ECU subsheath reconstruction resulted in satisfactory improvements in range of motion and grip strength. Although the mean improvements in these parameters were statistically significant, the clinical significance of these postoperative improvements remains to be defined.

**Type of study/level of evidence:**

Therapeutic, Level IV.

Extensor carpi ulnaris (ECU) subsheath injuries are increasingly recognized as an etiology of ulnar-sided wrist pain. The ECU tendon is stabilized within the sixth dorsal compartment by its fibro-osseous cartilaginous subsheath deep to the overlying extensor retinaculum (ER).^1^ Rupture of the ECU tendon subsheath is most commonly a result of forceful hypersupination, flexion, and ulnar deviation of the wrist, resulting in frank instability and painful ECU snapping with subluxation of the tendon outside the distal ulnar groove or apprehension with dynamic stress examination.^2^, ^3^, ^4^, ^5^ Subsheath insufficiency with or without ECU tendon instability often presents concurrently with intrinsic ECU pathology and ulnocarpal compartment injuries (such as triangular fibrocartilage complex [TFCC] and/or lunotriquetral interosseous ligament injuries). The coincidence of these injuries is rooted in the ECU “system” concept outlined by Graham,^6^ and further emphasizes the importance of identifying treatment options that can simultaneously address concomitant pathologies.

Diagnosis can often be made through a careful history and physical examination, demonstrating fullness/swelling along the length of the subsheath and tendon instability or apprehension. Magnetic resonance imaging (MRI) has been shown to improve diagnosis and provide valuable structural information, along with insight into concurrent tendinopathy and TFCC or other ulnocarpal compartment injuries.^7^ Dynamic ultrasound is also an effective method of confirming ECU tendon subluxation.^8^ Two different classification systems have been developed to describe various subsheath injuries.^9^^,^^10^

Nonsurgical treatment of acute ECU subsheath injuries is often successful.^1^^,^^6^^,^^11^ In recalcitrant cases requiring surgical intervention, there remains a lack of outcome data despite the variety of described operative techniques.^1^^,^^2^^,^^6^^,^^8^, ^9^, ^10^, ^11^, ^12^ Allende and Le Viet^9^ outlined their successful approach to ECU tendon and subsheath injuries requiring surgical correction: 4 cases of Inoue and Tamura^10^ type C (or Allende and Le Viet type B) subsheath injuries were treated with a transosseous suture approach, and 5 cases with true subsheath ruptures (Inoue and Tamura types A and B or Le Viet and Allende types C1 and C2) were treated with reconstruction using either a square piece of ER or a radially based ER sling approach. MacLennan et al^8^ then described their 21-patient series using a suture anchor-based approach while deepening the distal ulnar groove. Despite this, additional prospective outcome data are needed to elucidate optimal treatment approaches. We previously described our method using a radially based ER sling to treat ECU subsheath insufficiency.^5^^,^^13^ We believe that this approach is effective at improving function and allows simultaneous treatment of concurrent ulnocarpal compartment injuries. Based on our clinical experience, primary repair of the subsheath or surgical approaches that attempt to revise the osseous sulcus dimension may predispose it to complications, such as ECU stenosing tenosynovitis.

We demonstrate that the radially based ER sling is a safe and effective reconstructive strategy to treat ECU subsheath injuries. Additionally, in this retrospective series, we catalog and characterize the prevalence of concurrent wrist pathologies in patients with ECU subsheath injuries. We hypothesize that our radially based ER sling approach will result in statistically significant improvements in the wrist range of motion, grip strength, a full return to the preinjury level of activity, and minimal postoperative pain and complications in patients with ECU subsheath injuries.

## Materials and Methods

### Ethical approval

Our study was approved by the institutional review board at Newton-Wellesley Hospital. We retrospectively chart reviewed our hand-center experience of 33 patients (17 men; 16 women) who prospectively and consecutively underwent a radially based ER sling ECU subsheath reconstruction by 4 hand surgery-fellowship trained surgeons (D.E.R., M.L., M.B., M.A.V.) from April 2010 to April 2021. There were no exclusion criteria for this study. The independent variables addressed were age, sex, time to presentation/surgery, attempt at conservative management, preoperative imaging, presence of ECU snapping or apprehension with provocation (flexion, hypersupination, and ulnar deviation), and concurrent wrist injuries. The dependent variables addressed were range of motion values, such as flexion-extension arc (in degrees) and pronation-supination arc (in degrees), return to sport, and surgical complications.

### Patients

Patient demographics and injury characteristics are outlined in Table 1. Eight (35%) patients were collegiate or professional athletes. Surgery was performed on the right wrist in 16 (48.5%) patients and the left in 17 (51.5%) patients.

**Table 1:** Patient Demographics and Characteristics

Characteristic	Case Cohort (n = 33)
Age, median y (range)	44 (18–63)
Female, n (%)	16 (48.5)
Dominant wrist involved, n (%)	20 (60.6)
Etiology of injury, n (%)	
Sports	15 (45.5)
Weight-lifting	2 (6.1)
Household activities	8 (24.2)
Work-related activities	2 (6.1)
MVA	1 (3.0)
Atraumatic	5 (15.2)
Presence of ECU snapping, n (%)	
Frank snapping	10 (30.3)
Apprehension with provocation	12 (36.4)
ECU tenderness with palpation	11 (33.3)
Time between symptom onset and presentation, n (%)	
Acute (<2 wks)	4 (12.1)
Subacute (2–6 wks)	14 (42.4)
Chronic (> 6 wks)	15 (45.5)
Time between injury and surgery, median days (range)	199 (4–5885)
Nonsurgical management attempted, n (%)	29 (87.9)
Wrist arthroscopy performed, n (%)	29 (87.9)

MVA, motor vehicle accident.

**Figure 1:**
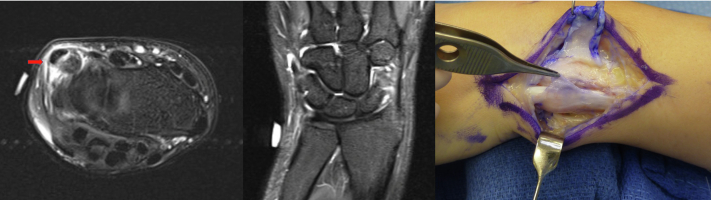
MRI and intraoperative findings showing a chronically attenuated Inoue and Tamura type C (Allende and Le Viet type B) ECU subsheath injury with concurrent ECU tenosynovitis.

**Figure 2:**
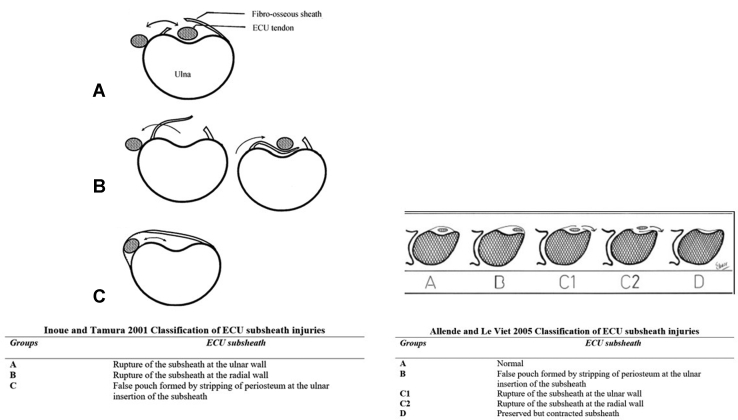
The Inoue and Tamura 2001 classification and the Allende and Le Viet 2005 classification of ECU subsheath tears.

Preoperative imaging included MRI in all 33 (100%) patients, plain-film radiographs in 24 (72.7%) patients, and ultrasound in 4 (12.1%) patients. All 33 (100%) patients had MRI findings of ECU subsheath injury (Fig. 1), whereas all 18 TFCC tears identified intraoperatively were also seen on MRI. Twenty-nine (87.9%) patients were treated conservatively prior to undergoing surgical intervention, whereas 4 (12.1%) patients with acute injuries (defined as <2 weeks) and severe pain elected for early surgical intervention despite being informed of nonsurgical treatment options. Nonsurgical management included anti-inflammatory medications, forearm immobilization in pronation with the wrist immobilized in a position of extension, and radial deviation for 4–6 weeks. For persistent painful snapping of the ECU, the use of cortisone injections was attempted to achieve painless snapping until repair/reconstruction, especially for in-season athletes. Twenty-six patients received cortisone injections as part of their nonsurgical management, with 4 athletes pursuing ECU subsheath reconstruction instead of steroids. Surgical treatment was otherwise indicated in refractory cases. The indication for concomitant wrist arthroscopy was foveal tenderness in combination with MRI findings of TFCC pathology. The median time from symptom onset to presentation was 61 days (range, 1–15 years), and the median time from symptom onset to surgery was 6.5 months (range, 4 days–16.1 years). Wrist arthroscopy was performed on 29 patients (87.9%; Table 1). Extensor carpi ulnaris subsheath injuries were graded according to both the Inoue and Tamura classification and the Allende and Le Viet classification (Fig. 2).^9^^,^^10^ Triangular fibrocartilage complex tears were graded according to the Palmer classification and scapholunate interosseous ligament tears were graded according to the Geissler classification.

Preoperative and postoperative grip strength and wrist range of motion measurements for flexion-extension arc and pronosupination arc were recorded by the senior surgeons (D.E.R., M.L., M.B., M.A.V.) or our hand therapist for each patient. Statistical analysis was conducted via a 2-tailed paired *t* test.

### Surgical technique

**Figure 3:**
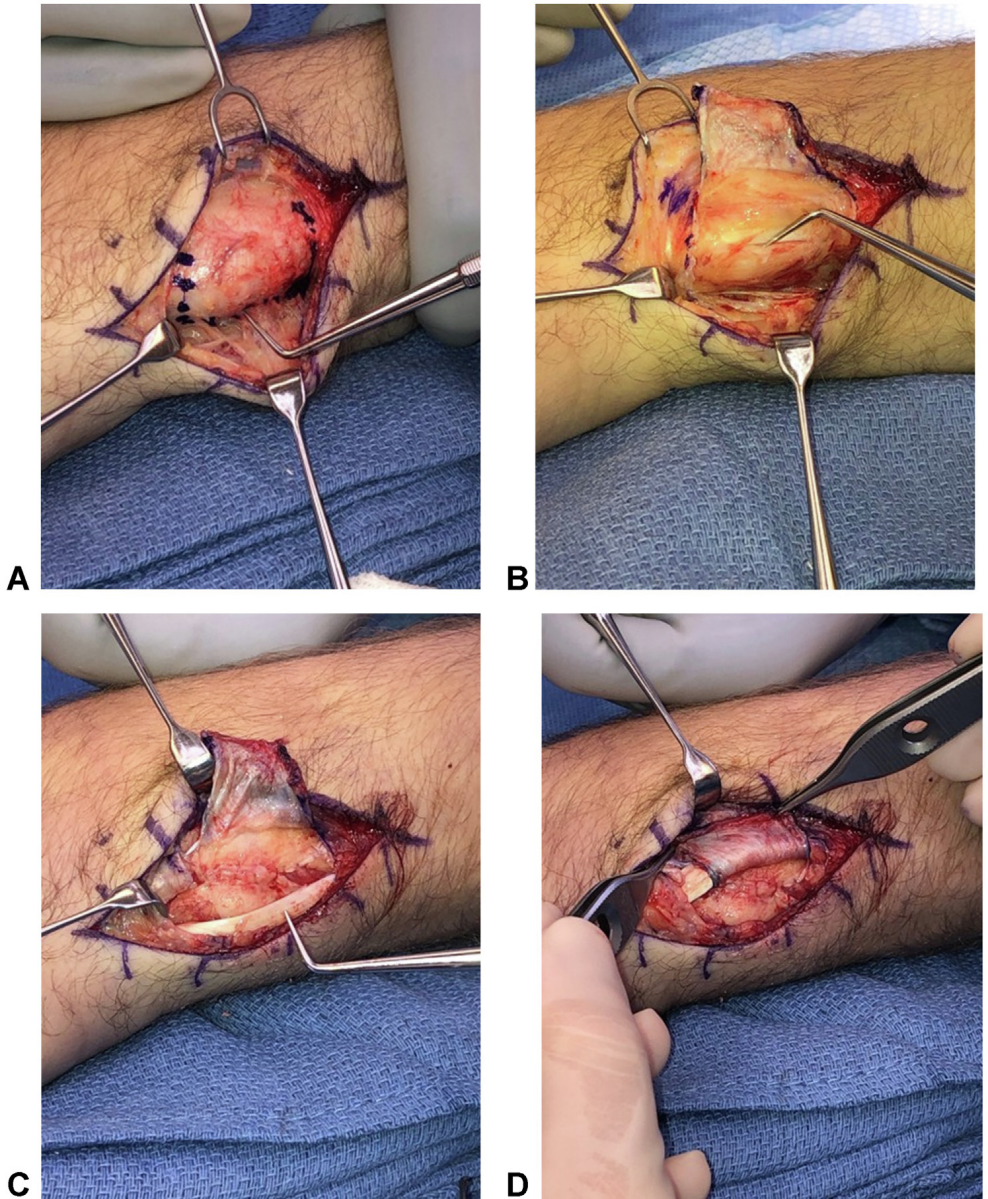
**A** Radially based extensor retinacular flap. **B** Following retinacular sling elevation, the acute tear of the fibrocartilaginous subsheath is seen with resultant ECU instability. **C** The dorsal-radial lip of the native osseous sulcus is subperiosteally coplaned flush with the floor of the sulcus to allow for smooth transitioning of the ECU during wrist and forearm motion. **D** Completed retinacular sheath reconstruction with smooth gliding and excursion of the ECU during pronosupination.

**Figure 4:**
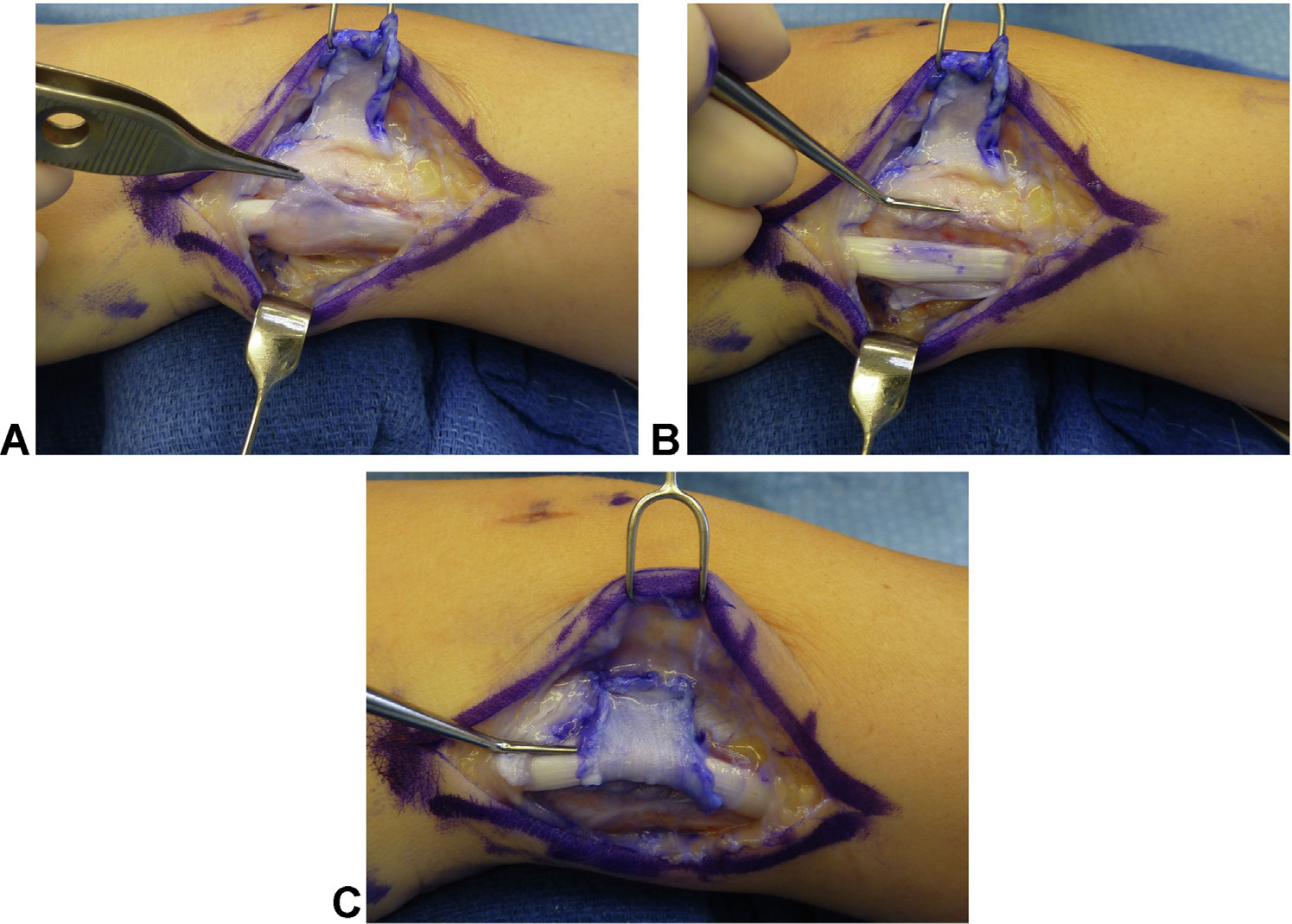
**A** The ECU has been translocated dorsal-radial during sling reconstruction. The extensor retinacular sling has been passed volar to the ECU and secured with multiple interrupted 2-0 absorbable monofilament sutures to the native retinaculum about the fifth extensor compartment. The subsheath has been approximated anatomically to the dorsal periosteum (pointer). **B** The dorsal-radial lip of the native osseous sulcus is subperiosteally coplaned flush with the floor of the sulcus to allow for smooth transitioning of the ECU during wrist and forearm motion. **C** Completed retinacular sheath reconstruction with smooth gliding and excursion of the ECU during pronosupination.

We previously described our surgical technique.^5^^,^^13^ A radially based ER sling is designed along the distal portion of the ER (Fig. 3). The retinacular flap is elevated as a separate layer from the ECU fibro-osseous sheath to the level of the extensor digiti quinti within the fifth extensor compartment. The underlying ECU subsheath is then inspected. The ECU is translocated dorsal-radial during the nonanatomic sling reconstruction. Then, the native volar-based fibro-osseous subsheath is approximated anatomically to the dorsal periosteum with buried interrupted sutures (3-0 undyed absorbable monofilament). This serves as a smooth interposed bed on which the sling and ECU can glide during pronosupination (Fig. 4). The ER sling is then passed volar to the ECU and secured with multiple interrupted 2-0 absorbable monofilament sutures to the native retinaculum about the fifth extensor compartment (Figs. 3, 4). Through a full range of pronosupination, the ECU is confirmed to track smoothly along the dorsal ulna without snapping or subluxation (Fig. 4).

### Postoperative follow-up

Following skin closure, a sugar tong long-arm orthosis with the elbow flexed, the forearm in pronation, and the wrist extended is applied. A long-arm cast with the forearm pronated and the wrist extended, or Munster orthosis, is used until approximately 4–6 weeks after surgery. When patients can achieve 45° of active pronation and supination, they transition out of the Munster orthosis and into a short-arm orthosis, followed by a progressive motion. Strengthening is initiated approximately 10 weeks after surgery. Sport-specific activities are allowed between 3 and 4 months after surgery in most cases. Major complications were defined as revision ECU subsheath surgery, recurrent ECU insufficiency, or nerve palsies lasting >6 weeks. Minor complications were defined as wound complications, transient neuritis, or other injuries stemming from the surgery that required follow-up. These complications were assessed on a physical examination and/or through imaging if warranted. We do not routinely conduct postoperative MRIs unless there is an indication. The preinjury level of activity was assessed according to the patient’s stated ability to perform common household chores, work responsibilities (typing, manual labor, and other tasks), and recreational/competitive sports activity.

## Results

In our prospective consecutive cohort, there were no cases of recurrent ECU subsheath insufficiency in our cohort, and there were no major complications following surgery. Minor complications included transient neuritis of the dorsal sensory nerve (n = 1). At the latest follow-up, there was no frank ECU instability or apprehension with a provocation on clinical examination. One elite athlete required secondary surgery for recurrence of grade 1B TFCC tear 7 months after surgery, although their ECU remained stable on examination and imaging. There was a statistically significant increase in grip strength and both wrist flexion-extension arc and pronosupination arc (*P* < .05) after surgery (Table 2). The mean time to unrestricted return to sports was 97.3 ± 19.7 days for the elite athletes in this study. All patients returned to their preinjury level of activity. The median follow-up was 9.2 ± 2.3 months.

**Table 2:** Wrist Range of Motion and Pain Before and After Radially Based Extensor Retinacular Sling Reconstruction of the ECU Subsheath

Variable	Mean Preoperative Value	Mean Postoperative Value	*P* Value
Pain on 0–10 scale, mean (SD)	Not recorded	0.39 (0.6)	N/A
Wrist flexion-extension arc° (SD)	128 (22)	136 (18)	.02
Wrist pronosupination arc° (SD)	155 (15)	162 (11)	.03
Grip strength, lbs (SD)	75 (24)	79 (21)	.01

Concurrent wrist pathologies found on preoperative imaging and/or wrist arthroscopy are listed in Table 3. Nineteen (82.6%) of 23 ECU subsheath injuries were type C (according to Inoue and Tamura^10^) or type B (according to Allende and Le Viet^9^), 2 (8.7%) of 23 were type A (according to Inoue and Tamura) or type C1 (according to Allende and Le Viet), one (4.3%) of 23 were type B (according to Inoue and Tamura) or type C2 (according to Allende and Le Viet). One (4.3%) of 23 had components of both types C and B (according to Inoue and Tamura) or types C1 and C2 (according to Allende and Le Viet). Although the remaining 10 patients did have an ECU subsheath injury, the specific injury classification was not recorded on chart review.

**Table 3:** Concurrent Wrist Injuries Presenting With ECU Subsheath Injuries

Concurrent Wrist Injuries	Case Cohort (n = 33)
Intrinsic extensor carpi ulnaris tendinopathy, n (%)	15 (45.4)
ECU tenosynovitis, n (%)	19 (57.6)
TFCC pathology, n (%)	20 (60.1)
Class 2A tear	8 (24.2)
Class 2C tear	1 (3.0)
Class 2D tear	2 (6.1)
Class 1A tear	3 (9.1)
Class 1B tear	1 (3.0)
Class 1D tear	2 (6.1)
Synovitis	3 (9.1)
Ulnocarpal synovitis, n (%)	20 (60.6)
Scapholunate interosseous ligament tear	3 (9.1)
Grade 1	2 (6.1)
Grade 2	1 (3.0)
Lunotriquetral interosseous ligament tears, n (%)	2 (6.1)
Extensor digiti quinti tenosynovitis, n (%)	3 (9.1)
Split tears requiring tubularization, n (%)	5 (15.2)
Split tears requiring palmaris longus autograft, n (%)	3 (9.1)
Ulnar impaction syndrome, n (%)	1 (3.0)
Distal radial ulnar joint instability, n (%)	1 (3.0)

Triangular fibrocartilage complex tears graded according to the Palmer classification. Scapholunate interosseous ligament tears graded according to the Geissler classification. For longitudinal split tears <50%, we perform tubularization. For split tears >50%, we use a palmaris longus autograft for repair.

## Discussion

There have been a number of described operative techniques for the treatment of ECU subsheath injuries. However, there is a lack of reported surgical outcomes, making it difficult to determine the optimal surgical approach. We performed the largest prospective consecutive cohort analysis to date. Our radially based ER sling approach to ECU subsheath reconstruction in 33 patients resulted in satisfactory outcomes and no major complications or recurrence of ECU subsheath insufficiency. Grip strength, wrist flexion-extension, and pronosupination arcs showed small improvement with minimal pain after surgery, although this is of unknown clinical significance. All patients returned to their preinjury level of activity, and athletes involved in our study were able to return to sport without restrictions in a quick timeframe without any recurrence of ECU subsheath insufficiency.

Our secondary objective was to characterize the prevalence of concurrent wrist pathologies in our cohort. Previous studies have not highlighted the coincidence of concurrent wrist pathologies. Allende and Le Viet^9^ also found that pure isolated tendon ECU tendon or subsheath pathologies are rare. They identified 11 (40.7%) TFCC tears and 4 (14.8%) lunotriquetral ligament tears. In our experience, ECU subsheath injuries most commonly presented with other ulnocarpal compartmental injuries, with only 2 (6.1%) cases of isolated ECU insufficiency. Extensor carpi ulnaris tenosynovitis, ulnocarpal synovitis, and/or TFCC pathology were present in most patients in our cohort, as outlined in Table 3. We identified 18 (54.5%) TFCC tears. We found that the majority of TFCC tears in our cohort were class 2A chronic degenerative tears, consistent with the chronic type C (according to Inoue and Tamura^10^) or type B (according to Allende and Le Viet^9^) subsheath insufficiency injuries observed in our cohort.

Although ECU instability can be asymptomatic, frank physical examination findings or subluxation/dislocation on imaging is rare in asymptomatic patients.^15^ Also, 66.7% of patients in our cohort had frank snapping or apprehension with a provocation on the examination with MRI findings of subsheath insufficiency and disruption. In contrast, the remaining 33.3% had subacute or chronic subsheath tears with clear findings of ECU tenderness in combination with MRI evidence. Instability was also confirmed on intraoperative examination. In addition, Sato et al^16^ illustrated how examination maneuvers are not always definitive in symptomatic patients, with ECU snapping being less prominent in the nonacute setting. Extensor carpi ulnaris tendon instability is also a well-documented source of ulnar-sided wrist pain, and chronic ECU subsheath attrition may eventually cause symptomatic tendon instability and tears, thus requiring surgical correction if nonsurgical management fails.^1^^,^^4^, ^5^^,^^10^^,^^17^ We acknowledge that it may be difficult to determine if ECU subsheath injury is the sole cause of pain. However, postoperative clinical examination showed no recurrence of snapping or apprehension with provocation in a cohort that functionally improved after ECU subsheath reconstruction.

Given the coincidence of ECU pathologies and ulnocarpal compartment injuries, we perform wrist arthroscopy prior to ECU reconstruction for both diagnostic purposes and treatment of ulnocarpal lesions, such as TFCC injuries. The 6R or 6U portal site(s) may be incorporated into the lazy-S incision used for ECU subsheath reconstruction in our approach. In the 4 patients who did not receive wrist arthroscopy, they had no signs of TFCC or distal radial ulnar joint pathology on examination or imaging. However, all had intrinsic tendon pathology or tenosynovitis, and the remaining patient had a rare isolated ECU subsheath tear and no concurrent injury. Our approach facilitates treatment of concurrent ECU tenosynovitis or longitudinal split tears, with elevation of the ER allowing for inspection and treatment of ECU tendon pathology while limiting the potential for stenosing tenosynovitis seen in primary subsheath repair. Results in these 4 patients were similar to the remainder of our cohort, demonstrating that our radially based technique with elevation of the ER plays a large role in treating concurrent pathology independent of wrist arthroscopy. Altogether, our approach allows the treatment of concurrent wrist injuries and may explain the lack of secondary surgeries in this cohort.^8^

MacLennan et al^8^ described their 21-patient series using an anatomical suture anchor repair approach while deepening the distal ulnar groove. Although the authors report promising results, including a significant reduction in pain and Disabilities of the Arm, Shoulder and Hand scores, this approach precludes the treatment of concomitant ulnocarpal compartment injuries and may also predispose to stenosing tenosynovitis of the ECU, and deepening the distal ulnar groove may increase the intrinsic force on the ECU tendon predisposing to suture anchor failure and ulna fractures.^4^^,^^6^^,^^16^

Allende and Le Viet^9^ previously described their approach to ECU tendon and subsheath injuries in their 27-patient cohort. For Allende and Le Viet type B subsheath injuries, they used a transosseous suture repair approach. For Allende and Le Viet types C1 and C2 subsheath injuries, they performed subsheath reconstructions using a square piece of ER or a radially based ER sling approach. In addition to a palmaris longus autograft, they performed subsheath reconstructions in Allende and Le Viet type D injuries. While 15 patients had isolated tendon pathology and no subsheath injury, 5 (26%) patients had restricted wrist range of motion and 7 had >30% reduction in grip strength. In contrast, no patients in our cohort had a restricted wrist range of motion or more than a 30% reduction in grip strength.

Fram et al^14^ also used an ER sling approach and reported on their 11-patient cohort, which consisted of 7 athletes. They reported improvement in pain, patient-reported subjective outcomes, and demonstrated overall patient satisfaction with the procedure. We have similarly found that patients are often very satisfied following our similar ER sling approach. In addition to our larger sample size, the restoration of the range of motion, and function in this series supports that radially based ER sling reconstruction facilitates recovery from ECU subsheath insufficiency. We also found a similar time to unrestricted return to sport in the athletes in our study, further illustrating that the radially based ER sling approach is effective in high-level athletes. Moreover, all 4 acute subsheath injuries in our cohort were in elite athletes, suggesting that our reconstructive approach is an acceptable acute alternative to acute subsheath repair and allows an expedited return to sport.

A number of other studies have described various surgical approaches to ECU subsheath insufficiency, albeit without surgical outcome data. Although Graham^6^ advocates for an ulnar-based ER flap out of concern for soft tissue and rim failure in radially based approaches, persistent or recurrent instability was not demonstrated in our cohort. Although authors have described repair techniques aimed at restoration of the linea jugata as a labral barrier to recurrent ECU instability, we favor formal subsheath reconstruction and use the native residual subsheath to fill the native osseous sulcus to prevent the ECU from riding over the residual dorsal lip into the sulcus once the ECU is translocated.^18^ Eckhardt and Palmer^3^ used a free graft of ER to reconstruct the ECU subsheath. Inoue and Tamura^10^ used a combination of surgical approaches in their 12-patient cohort, depending on the type of ECU subsheath injury. They used an ER-based approach for type A, a direct suture approach for type B, and a reattachment of the periosteum for type C injuries. We demonstrated that our radially based ER sling approach was effective at treating all Inoue and Tamura subtypes of ECU subsheath injuries in our cohort.

There are several limitations to our prospective study. It is a single-center study without a comparison subgroup. A lack of patient-reported outcomes prior to surgery limited our ability to report on subjective outcomes. However, our outcomes compare favorably to all studies looking at surgical outcomes following surgical correction of the ECU subsheath to date. The variable time to presentation and postoperative follow-up may also contribute to our findings, although patients were instructed to return if symptoms recurred and all had returned to daily life activities at the time follow-up was made optional. Despite these limitations, we believe that this study contributes substantially to the current understanding of the surgical treatment of ECU subsheath insufficiency. Future studies should continue to focus on surgical outcome reporting and subgroup analyses.
